# OTEH-4. Deeper insight into intratumoral heterogeneity by MRI and PET-guided stereotactic biopsies from glioblastoma patients

**DOI:** 10.1093/noajnl/vdab070.043

**Published:** 2021-07-05

**Authors:** Atul Anand, Jeanette Krogh Petersen, Mark Burton, Martin Jakob Larsen, Lars van Brakel Andersen, Dylan Scott Lykke Harwood, Christian Bonde Pedersen, Frantz Rom Poulsen, Peter Grupe, Torben A Kruse, Mads Thomassen, Bjarne Winther Kristensen

**Affiliations:** 1 Department of Pathology, Odense University Hospital, Odense, Denmark; 2 Department of Clinical Research, University of Southern Denmark, Odense, Denmark; 3 Department of Clinical Genetics, Odense University Hospital, Odense, Denmark; 4 Clinical Genome Center, Department of Clinical Research, University of Southern Denmark, Odense, Denmark; 5 Department of Clinical Medicine and Biotech Research and Innovation Center (BRIC), University of Copenhagen, Copenhagen, Denmark; 6 Department of Pathology, The Bartholin Institute, Rigshospitalet, Copenhagen University Hospital, Copenhagen, Denmark; 7 BRIDGE (Brain Research - Inter Disciplinary Guided Excellence), Odense University Hospital and University of Southern Denmark, Odense, Denmark; 8 Department of Neurosurgery, Odense University Hospital, Odense, Denmark; 9 Department of Nuclear medicine, Odense University Hospital, Odense, Denmark

## Abstract

Glioblastoma is one of the most aggressive cancers, but the molecular evolution is still not fully understood. We used PET imaging combined with deep sequencing of glioblastoma biopsies at both the RNA and DNA levels to get a deeper insight into molecular evolution. In the clinical setting, PET imaging provides information about metabolically active tumor areas, but the molecular interpretation is unclear. Our primary objective was to perform an intratumoral spatial comparison of biopsies from potentially aggressive and less aggressive areas in glioblastomas according to PET scans. Additionally, tissue from the tumor periphery was included. We used MRI, ^11^C-methionine(MET) PET, and ^18^F-FDG PET was used in combination to obtain a series of neurosurgical stereotactic biopsies from tumor areas with high MET and ^18^F-FDG uptake (hotspot), low MET and ^18^F-FDG uptake (coldspot), as well as tumor periphery of six glioblastoma patients that were processed for whole genome, exome, and transcriptome sequencing. Differential gene expression and gene ontology analysis showed that hotspots were enriched in gene sets associated with DNA replication, cell cycle, and ligand receptor interaction. Genome and exome analysis suggested hotspots and coldspots to have similar mutational profiles. However, a limited number of hotspot-specific mutations and fusion transcripts indicated that hotspot tumor cells developed from coldspot cells and point at the potential role of hotspot driver genes in glioblastoma. Our findings reveal that hotspots in glioblastomas represent a more advanced stage of molecular evolution than coldspots.

